# Optimizing the Diagnosis of Ureteral Complications After Kidney Transplantation Using Retrograde Multidetector Computed Tomography Urography: A Case Series

**DOI:** 10.7759/cureus.103538

**Published:** 2026-02-13

**Authors:** Maria SidiropouIou, Androniki Gkana, Athanasios Kofinas, Athanasia Anastasiou

**Affiliations:** 1 Department of Radiology, Ippokrateio General Hospital of Thessaloniki, Thessaloniki, GRC; 2 Department of Transplantation Surgery, Ippokrateio General Hospital of Thessaloniki, Thessaloniki, GRC

**Keywords:** case series, ct urography, kidney transplantation, multidetector computed tomography urography, retrograde multidetector computed tomography urography, retrograde urography, ureteral complications, urinoma

## Abstract

Ureteral complications remain a significant cause of morbidity following kidney transplantation and may threaten graft survival if not diagnosed promptly. Retrograde multidetector computed tomography (MDCT) urography has emerged as a valuable imaging modality for detailed evaluation of the transplant ureter. This case series highlights the role of retrograde MDCT urography in the assessment of post-transplant ureteral complications, particularly urine leaks and ureteral obstructions, in patients with inconclusive or non-diagnostic ultrasound findings. In the presented cases, retrograde MDCT urography accurately localized the site and extent of ureteral leaks, facilitated timely therapeutic decision-making, and contributed to preservation of graft function. The technique provided high spatial resolution and comprehensive anatomical visualization of the collecting system and ureter. These observations support the role of retrograde MDCT urography as a reliable and effective adjunct imaging modality of the diagnostic algorithm for renal transplant recipients with suspected urological complications.

## Introduction

Kidney transplantation is the treatment of choice for end-stage renal disease, yet it is associated with urological complications, including urine leaks, ureteral obstruction, vesicoureteral reflux, and lymphocele formation [1-3]. Urine leakage, occurring in 1.2-8.9% of cases, is a significant complication that may arise from surgical technique errors, ischemia-induced distal ureteral necrosis, or extrinsic compression [4,5]. Early and accurate diagnosis of these complications is essential for preventing graft loss and improving patient outcomes [3]. While ultrasound is often used for initial graft evaluation, its limitations necessitate the use of more sensitive imaging modalities, such as CT urography [6-8]. This study explores the role of retrograde MDCT urography in diagnosing ureteral complications after kidney transplantation. The cases in this article were previously presented as a poster at the European Congress of Radiology (ECR) on February 26, 2025.

## Case presentation

Technique for retrograde CT urography

In a series of cases involving post-kidney transplantation urinary complications, MDCT imaging without intravenous contrast was utilized. All patients underwent MDCT using a 64-slice General Electric Optima CT660 CT scan machine (GE HealthCare, Boston, MA). A 5-7% diluted iodinated contrast solution (Iopromide-Ultravist-370 (Bayer AG, Leverkusen, Germany)), prepared with saline, was infused through a Foley catheter to evaluate transplant ureter and ureteral stents, leaks, drainage, and stenosis. Approximately 240 mL of contrast fluid, divided into four 60 mL portions, was used to fill the bladder and transplant ureter, adhering to a sensitivity cutoff of 250 mL [5]. Adjustments were made based on patient tolerance or early detection of abnormalities. MDCT imaging was performed from the iliac crests to the lesser trochanters using 1.25 mm-thick sections and included axial views, sagittal, and coronal reconstructions. Patients were scanned in either the supine or prone position, with post-drainage imaging typically unnecessary. All four kidney transplant cases demonstrated urinary leaks at the urinary tract anastomosis, leading to urinoma formation in some cases. Three patients required surgical repair along with conservative treatment, while another required MDCT-guided drainage before surgery. 

Case one

**Figure 1:**
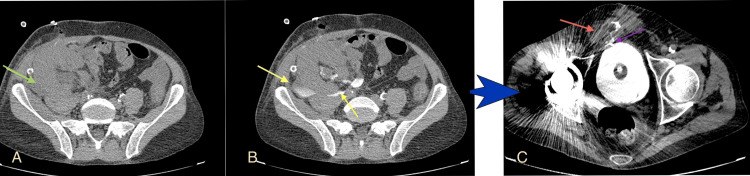
Leak at the level of the anastomosis of the renal pelvis and the native ureter (A) Axial multidetector computed tomography (MDCT) image before contrast administration reveals a low-density fluid collection around the transplant kidney (green arrow); (B) Axial MDCT image after bladder infusion with diluted solution shows a high-density collection outside the collecting system (yellow arrows) and around the transplant; (C) Axial image in the same patient after the Politano-Leadbetter ureteric reimplantation shows the distal part of the ureteric stent (purple arrow) and a remaining leak, this time from the ureter-bladder anastomosis (red arrow), with fistula formation with the lower abdominal wall. Image source: Department of Radiology, Ippokrateio General Hospital of Thessaloniki. Permission obtained for reproduction.

This case involved a urine leak from the pyelo-ureteral anastomosis in the late post-operative period. A 59-year-old male kidney transplant recipient presented with pain and increased creatinine levels two weeks postoperatively. Retrograde MDCT urography confirmed a urine leak and fluid collection (Figures 1A, 1B).

Surgical repair with the Politano-Leadbetter technique [9] couldn’t manage the leak at first (Figure 1C), but along with conservative support with a Foley catheterization and urinary diversion, the leak gradually improved.

Case two

**Figure 2:**
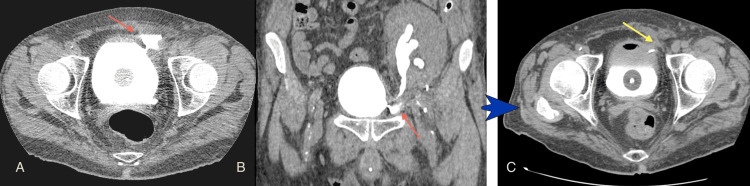
Urine leak of the anastomosis close to the bladder (A) Axial image and (B) Coronal multidetector computed tomography (MDCT) projection show contrast medium outside the urinary system at the entry of the ureter in the bladder (red arrows); (C) After conservative treatment, the follow-up retrograde MDCT urography shows no leakage around the ureteric stent (yellow arrow). Image source: Department of Radiology, Ippokrateio General Hospital of Thessaloniki. Permission obtained for reproduction.

This was a case of gradual urinoma formation. A 48-year-old male patient presented with dull pain at the site of the transplantation one month postoperatively. The patient developed a urinoma because of ureterovesical anastomosis leakage. MDCT urography revealed a high-density collection below the transplanted kidney and adjacent to the bladder. The patient improved with supportive care and ureteric stent placement (Figure 2).

Case three

**Figure 3:**
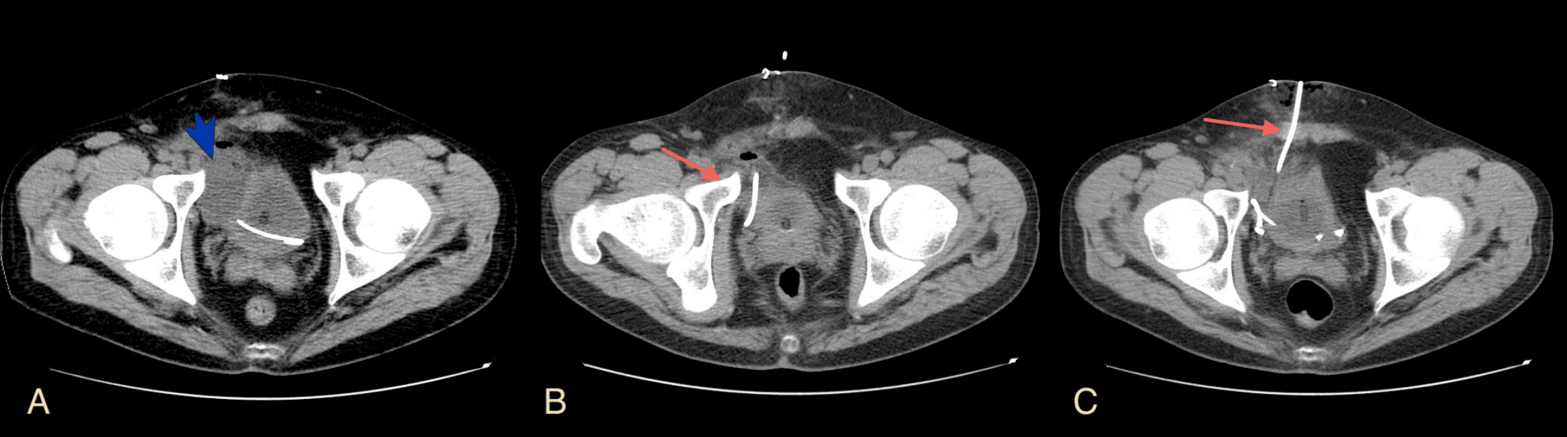
MDCT-guided placement of a drainage catheter in a urinoma one month after kidney transplantation (A) An axial multidetector computed tomography (MDCT) image without IV or retrograde contrast medium suggests a urinoma due to the low-density, round collection in contact with the bladder at the site of ureterovesical anastomosis (blue arrowhead). The bladder cavity contains the distal part of the ureteric stent and the Foley catheter cuff; (B, C) Axial MDCT images after the drainage catheter placement (red arrows). The urinoma was again confirmed by laboratory examination of the drained fluid. Image source: Department of Radiology, Ippokrateio General Hospital of Thessaloniki. Permission obtained for reproduction.

Τhis case involved a persistent urine leak from the lower part of the surgical trauma. A 46-year-old male patient experienced a persistent urine leak from the ureterovesical anastomosis, leading to small fistula formation with the lower abdominal wall. MDCT examination demonstrated fluid collection at the site of the anastomosis (Figure 3A).

**Figure 4:**
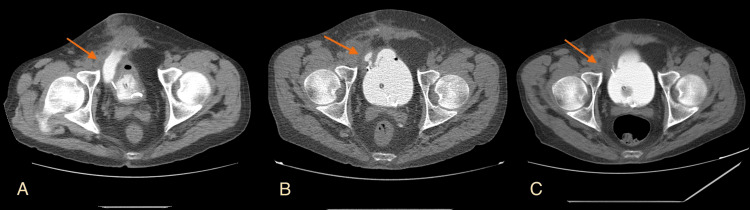
Urine leak after Politano-Leadbetter ureteric reimplantation Axial multidetector computed tomography (MDCT) urography images of the first (A), second (B), and third (C) postoperative months of the ureteric reimplantation show the residual leakage and gradual improvement along with conservative management (red arrows). Image source: Department of Radiology, Ippokrateio General Hospital of Thessaloniki. Permission obtained for reproduction.

**Figure 5:**
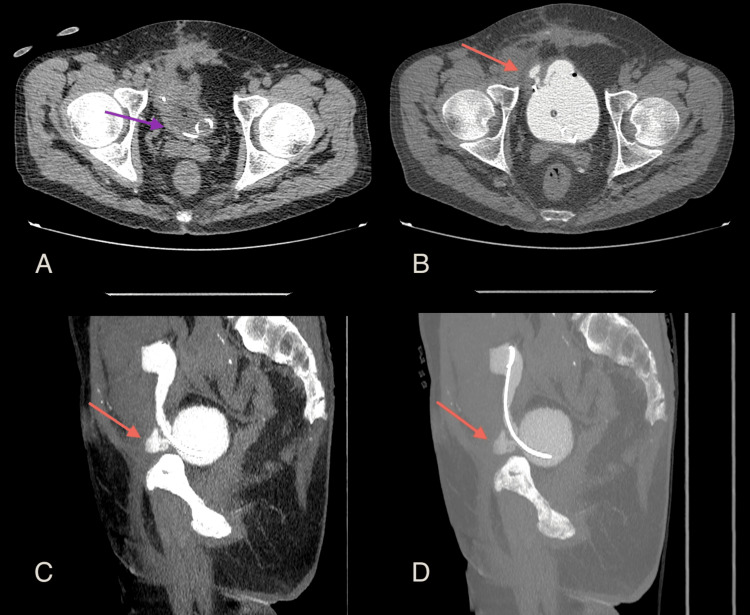
Urine leak of the ureterovesical anastomosis during the second postoperative month of the ureteric reimplantation Multidetector computed tomography (MDCT) urography (axial view) (A) before and (B) after retrograde contrast medium administration; (A) depicts the empty bladder, including the Foley catheter and the distal part of the ureteric stent (purple arrow); (B) indicates a collection of contrast medium outside the bladder at the anastomosis level (red arrow); (C) Sagittal MDCT projection outlines the urine leakage (red arrow); (D) Sagittal MDCT Maximum Intensity Projection (MIP) view depicts the leak (red arrow) and the ureteric stent. Image source: Department of Radiology, Ippokrateio General Hospital of Thessaloniki. Permission obtained for reproduction.

MDCT-guided placement of drainage in the urinoma was followed (Figures 3B, 3C). Because of the continuous leak, the patient eventually underwent a Politano-Leadbetter ureteric reimplantation, resulting in a successful resolution (Figures 4, 5).

Case four

**Figure 6:**
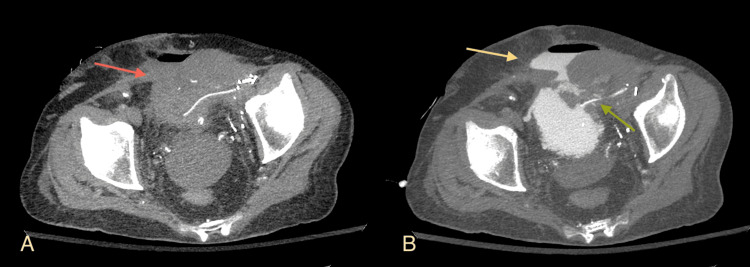
Double urine leak in a patient with a history of previous leak after kidney transplantation and Boari flap surgery (A) Multidetector computed tomography (MDCT) Maximum Intensity Projection (MIP) axial projection before retrograde administration of contrast medium indicates a hypodense fluid collection containing air adjacent to the bladder, involving the anterior lower abdominal wall; (B) MDCT MIP axial projection after the retrograde administration of contrast medium. There is a hyperdense small leak around the ureteric stent (green arrow) and a larger collection containing fluid and air in the lower anterior abdominal wall due to leak from the upper wall of the bladder (yellow arrow). Image source: Department of Radiology, Ippokrateio General Hospital of Thessaloniki. Permission obtained for reproduction.

This patient was also a complicated leak case involving a double urine leak after the Boari flap surgery for a persistent urine leak after kidney transplantation. A 49-year-old male patient with a kidney transplant and prior Boari flap surgery for ureteral reconstruction developed a double urine leak. Retrograde MDCT urography identified one leak proximal to the ureterovesical junction and another in the anterior bladder wall (Figure 6).

Staged surgical correction was performed, and the patient recovered successfully.

All the four patients with ureteral complications after kidney transplantation were successfully diagnosed using retrograde MDCT urography without intravenous contrast, and demonstrated 100% detection of urinary leaks at the ureterovesical or pyelo-ureteral anastomosis, as summarized in Table 1. 

Three patients required surgical repair (two with Politano-Leadbetter reimplantation, one with Boari flap technique) combined with conservative management, while one patient (case two) achieved resolution with conservative care and ureteric stent placement alone. One patient (case three) underwent MDCT-guided percutaneous drainage prior to definitive surgery, illustrating the dual diagnostic and therapeutic capability of the technique. All patients ultimately achieved successful graft preservation and resolution of their complications (Table 1).

**Table 1:** Summary of the diagnostic evaluation, therapeutic procedure, and outcomes of the four patients MDCT: Multidetector computed tomography.

Case	Age/Sex	Presentation timeline	Retrograde MDCT urography findings	Diagnostic advantage	Management	Outcome
1	59 yrs; Male	Pain, elevated creatinine 2 weeks post-op	Urine leak with fluid collection clearly visualized on pre- and post-contrast images	Precise leak localization without IV contrast	Urinary diversion and Politano-Leadbetter reimplantation	Successful resolution
2	48 yrs; Male	Dull pain at transplant site 1 month post-op	Urinoma from ureterovesical anastomosis leak; high-density collection	Clear anatomic delineation of the leak site and relationship to adjacent structures	Conservative care	Successful resolution
3	46 yrs; Male	Persistent urine leak from surgical trauma 1 month post-op	Ureterovesical anastomosis leak with a small fistula to the lower abdominal wall	MDCT-guided percutaneous catheter placement followed by surgical planning	MDCT-guided drainage, Politano-Leadbetter reimplantation	Successful resolution after a staged approach
4	49 yrs; Male	Recurrent complications post-Boari flap (prior leak)	Double leak: one proximal to ureterovesical junction, one in anterior bladder wall	Detection of multiple simultaneous leaks	Boari Flap and further staged surgical correction	Successful recovery after a staged approach

The retrograde MDCT urography technique offered critical advantages in this transplant population. It eliminated the risk of contrast-induced nephrotoxicity by avoiding intravenous contrast administration in patients with compromised renal function, provided superior anatomic detail with thin-slice (1.25 mm) multiplanar reconstruction for precise leak localization, and utilized readily available equipment (standard 64-slice MDCT) with a simple bladder infusion protocol requiring only diluted contrast solution (5-7% Iopromide). This ease of implementation and immediate diagnostic yield enabled rapid clinical decision-making regarding conservative versus surgical management while maintaining excellent safety in high-risk patients.

## Discussion

Urine leakage typically occurs in the immediate or early post-transplant period, usually within the first three months [3,4]. Most leaks originate from the distal ureter, most frequently at the ureteroneocystostomy site [2,3]. A key predisposing factor is devascularization of the distal ureter during organ retrieval, which compromises its blood supply [2,4]. Inadequate surgical technique during ureteroneocystostomy may further contribute by resulting in an anastomosis that is not watertight [2]. In the absence of overt technical errors, ischemia-related distal ureteral necrosis is considered the predominant mechanism underlying early ureteral complications following kidney transplantation [3].

Clinically, urine leaks may present with graft-site pain or swelling, rising serum creatinine levels, oliguria, and signs of systemic infection. In the immediate postoperative period, urine leakage may be detected through surgical drains or from the wound itself [3]. Extravasated urine can also track into the retroperitoneum, pelvis, presacral region, or scrotum, or become encapsulated, forming a urinoma. These collections may exert mass effect on vascular structures or the urinary outflow tract, leading to impaired graft function. Additionally, urine leaks increase the risk of surgical site infection, potentially resulting in perinephric abscess formation and delayed wound healing [3].

Retrograde MDCT urography is an effective imaging technique for detecting urinary leaks and obstructions following kidney transplantation. Compared to ultrasound and voiding cystography, MDCT urography provides superior visualization of the urinary tract, with high sensitivity for early-stage urine and small leaks [7] and more detailed detection of ureteral strictures and reflux [4]. The use of diluted contrast solutions enhances visualization of the collecting system while minimizing patient discomfort [5]. Non-contrast MDCT can also differentiate hematoma from urinoma, as the former is of high density (60-90 Hounsfield Units) [4]. When intravenous contrast medium is used, an MDCT scan helps differentiate between a urinoma, an abscess, and a lymphocele [3]. 

**Figure 7:**
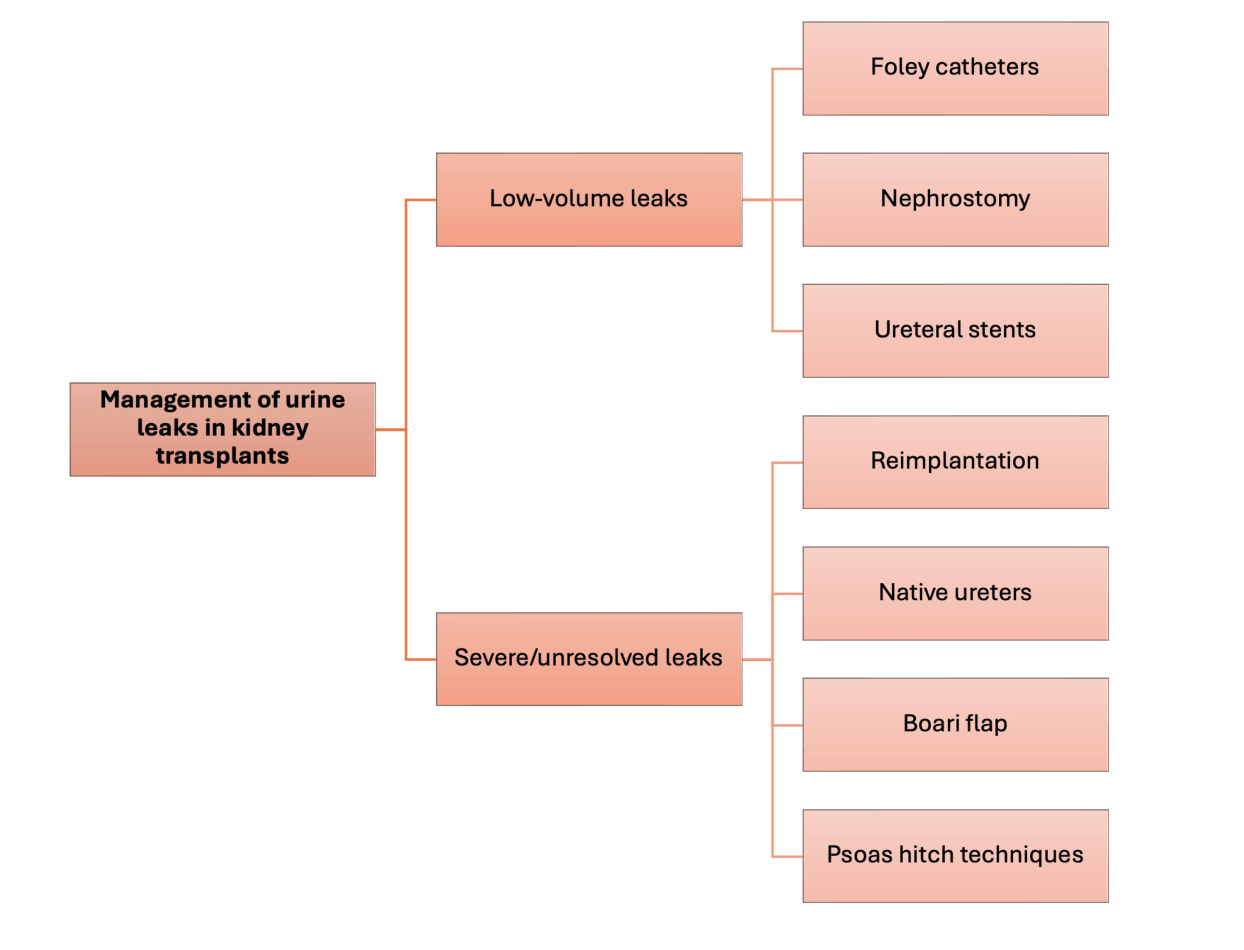
A simplified management diagram of urine leaks in kidney transplants, depending on severity. Image credit: Created by Gkana A. based on published literature [2-4] using Microsoft PowerPoint for Mac (version 16.105.2; Microsoft Corp., Redmond, WA, USA) and Preview (version 11.0; Apple Inc., Cupertino, CA, USA).

Management of urine leaks depends on the severity of the leak as described through imaging. Low-volume leaks are generally managed conservatively through maximum urinary decompression (Figure 7) [2].

Severe or unresolved leaks with conservative management require surgical intervention for urinary tract reconstruction (Figure 7) [2-4]. Urgent percutaneous drainage, often with CT guidance, is indicated for infected fluid collections or obstructions. CT-guided percutaneous drainage can be performed at the time of diagnosis, reducing the need for repeat imaging and interventions [2,6]. The Lich-Grégoir technique is favored for ureterovesical anastomosis due to its lower leakage rates [1,3,4]. In Figure 7, a prompt management guide is proposed to ensure favorable outcomes despite potential morbidity. 

## Conclusions

Retrograde MDCT urography represents a critical diagnostic tool for detecting ureteral complications following kidney transplantation, with demonstrated 100% sensitivity for identifying urine leaks in this case series. Its key advantages include elimination of nephrotoxicity risk through avoidance of intravenous contrast, superior anatomic visualization with thin-slice multiplanar reconstruction, and the ability to guide both diagnostic and therapeutic interventions, such as CT-guided drainage. The technique enabled early diagnosis and appropriate selection of management techniques in all four cases, contributing to successful graft preservation. As urological complications in kidney transplant recipients represent a significant cause of graft dysfunction, the availability of a safe, accurate imaging modality is essential for optimizing transplant outcomes. Based on these findings, retrograde MDCT urography should be integrated into standard post-transplant imaging protocols when urological complications are suspected, particularly when ultrasound findings are inconclusive or when precise anatomic localization is required for surgical planning. Further prospective studies with larger patient cohorts would help establish standardized protocols and validate routine use in post-transplant monitoring.
